# Supplementary material for: Physicochemical properties-based hybrid machine learning technique for the prediction of SARS-CoV-2 T-cell epitopes as vaccine targets
Source: PeerJ Comput Sci. 2024 Apr 25;10:e1980. doi: 10.7717/peerj-cs.1980 (PMC11057572; doi:10.7717/peerj-cs.1980)
Supplement: Supplemental Information 1 [file peerj-cs-10-1980-s001.docx]

Supplementary Table S1 **Optimal feature sets by different FS techniques**

| **FS Technique** | **Optimal Feature set** | **Count** |
| --- | --- | --- |
| HcS | F8_13,F8_27,F8_14,F7_13,F7_32,F8_3, F11_3,F7_45,F9_2,F7_36,F7_8,F8_7,F8_24,F4,F8_15,F8_43,F8_1,F8_25,  F7_33,F7_7,F8_6,F8_16,F7_6,F7_39,F8_42,F7_41,F8_29,F7_43,F7_1,F7_20,F7_22,F11,F7_21,F8_11,F8_37,F7_17,  F7_26,F7_34,F8_31,F7_31,F7_14,F7_11,F7_27,F8_8,F7_42,F7_19,F8_23,F7_30,F7_38,F7_23,F5_2,F8_38,F7_28,  F7_44,F8_32,F7_9,F7_29,F8_21,F7_3,F7_25,F11_7 | 50 |
| FwS | F8_47,F8_4,F7_22,F8_9,F1,F8_48,F8_18,F8_17,F8_31,F9_3,F8_2,F8_28,F7_27,F8_26,F8_46,F8_40,F8_44,F7_24,  F8_39,F7_18,F8_30,F9_9,F8_41,F2,F8_45,F9_1,F5_1,F11_7,F7_26,F3,F8_33,F7_43,F7_42,F10,F9_6,F7_23,F7_25,F8_36,  F7_40,F7_17,F7_29,F7_28 | 41 |
| BwS | F9_5, F1,F9_4,F8_35,,F7_11,F11_13,F7_2,F8_49,F7_14,F7_13,F7_4,F4,F7_38,F7_24,F7_12,F7_37,F7_15,F7_10,  F7_5,F8_5,F6_1,F2,F3,F8_19,F7_8,F8_34,F7_16,F7_33, F7_40,F7_1,F8_12,F7_39,F7_30,F7_32,F7_35,F7_19,F9_7,  F4F7_34,F7_41,F7_18,F7_21,F6_2,F8_10,F8_20,F7_31,F8_22 | 47 |
