# Supplementary material for: Physicochemical properties-based hybrid machine learning technique for the prediction of SARS-CoV-2 T-cell epitopes as vaccine targets
Source: PeerJ Comput Sci. 2024 Apr 25;10:e1980. doi: 10.7717/peerj-cs.1980 (PMC11057572; doi:10.7717/peerj-cs.1980)
Supplement: Supplemental Information 2 [file peerj-cs-10-1980-s002.docx]

**Supplementary Note S1** Description of Classifiers used

1. ***Decision Tree (DT):*** The DT algorithm uses a greedy approach to divide the feature space recursively using a binary partitioner. At the bottommost partition (leaf), the same label is predicted. In order to achieve this, a partition is selected in a greedy manner from a group of potential splits by picking the optimal split that maximizes the information gain at a given node in the tree.
2. ***Random Forest (RF):*** The RF algorithm is a classification approach that employs an ensemble of decision trees with an added layer of randomness through the bagging method. At each node in the RF algorithm, the best predictor is selected from a set of predictors that are randomly chosen for that node.
3. ***Neural Network (NN):*** The NN algorithm employed in this research is a single-layer feed-forward backpropagation network. NNs are composed of interconnected information processing units that utilize an activation function to transform inputs into outputs. In backpropagation networks, the training process usually involves using a delta rule to determine the difference between the desired and actual outputs, which is also known as the error. This error is then propagated backwards to all the units in the network, and the weights are adjusted at each connection to enhance the overall performance.
